# Irving Gottesman: American psychologist and behaviour geneticist who radically changed traditional views of schizophrenia

**DOI:** 10.1192/pb.bp.116.055582

**Published:** 2017-04

**Authors:** Peter McGuffin

**Figure:**
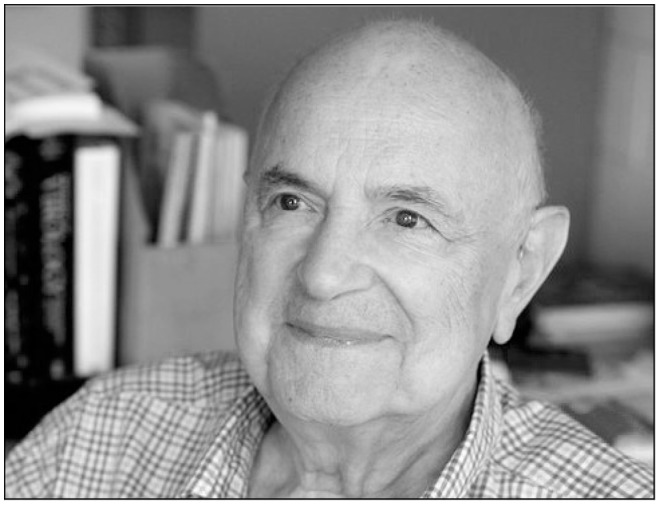
Irving Gottesman used a register of twins at the Maudsley and Bethlem Royal hospitals in London for his study of schizophrenia. Photograph: Lisa Miller, University of Minnesota/theguardian.com

The research and writings of the clinical psychologist and behaviour geneticist Irving Gottesman, who has died aged 85, radically changed the way psychologists and psychiatrists think about schizophrenia and, more generally, about what Irv called the ‘origins of madness’ (which was the subtitle of his 1991 book Schizophrenia Genesis).

His path to becoming a dominant figure in psychiatric genetics began at an international conference in 1961 when, as a psychology lecturer at Harvard University, he was introduced to Eliot Slater, director of the Medical Research Council (MRC) psychiatric genetics unit at the Institute of Psychiatry in London, and the doyen of psychiatric genetics of his day.

Slater agreed that Irv might join his unit provided he brought his own funding. Irv duly won a three-year grant from the US National Institutes of Health (NIH) and arrived in London in 1963. Slater was an austere and imposing figure and the building that housed his unit was equally austere, a makeshift postwar prefabricated building, affectionately known by staff as ‘the hut’, on the fringe of the campus of the Maudsley hospital in Camberwell. Although its physical environment was poor, the unit had much intellectual capital, provided by Slater himself, his deputy director Valerie Cowie, a psychiatrist trained in the new techniques of cytogenetics, and a brilliant if self-effacing senior research assistant, Jerry Shields.

The hut also housed another world-class asset, the Maudsley Twin Register. Begun by Slater in 1948, it contained the names of all patients entering the Maudsley and Bethlem Royal hospitals who had been born a twin. Irv's successful NIH proposal was for a study of schizophrenia using the register and Slater paired him with Shields to carry out the work.

The principle of the classic twin method is straight-forward. Identical or monozygotic (MZ) twins share all their genes, whereas fraternal or dizygotic (DZ) twins share half their genes. MZ and DZ twins usually share the environment in which they are raised. Therefore if a disorder such as schizophrenia shows greater co-occurrence (‘concordance’) in MZ versus DZ twins this is evidence of a genetic effect. Similarly, absence of 100% concordance in MZ twins is evidence of environmental effects. The Gottesman-Shields Twin Study (1967) clearly confirmed the proposition that both genes and environment play a role in schizophrenia (at a time when theorists in the US and Britain were blaming parents, particularly mothers, for ‘causing’ the disorder).

One of Irv and Jerry's major contributions was to propose a plausible mode of inheritance for schizophrenia. One of the big puzzles about familial common diseases at the time was that none (including physical disorders such as heart disease, diabetes, some cancers) showed the simple ratios of affected:unaffected within families predicted by Mendel's laws.

The most widely accepted solution for schizophrenia was Slater's model invoking the idea of a dominant gene with ‘incomplete penetrance’ (some people carry the gene but do not show the disorder). Irv and Jerry boldly proposed an alternative polygenic model, derived from the work of the Edinburgh mathematical geneticist DS (Douglas) Falconer, in which liability to develop schizophrenia has a normal ‘bell-shaped’ distribution in the population (like height or weight) contributed to by many genes. But, unlike height or weight, there is a threshold effect, so that only the 1% or so of the population with the highest liability show the disorder.

The Gottesman-Shields polygenic model of schizophrenia eventually gained ascendancy, even though the final clinching piece of evidence emerged only in 2014 with the publication of a huge genome-wide molecular study of tens of thousands of subjects showing that more than 100 genes are involved.

Another far-reaching conceptual innovation was their idea of ‘endophenotypes’. Irv and Jerry proposed in their 1972 book, Schizophrenia and Genetics, that the genetic basis of psychiatric disorders could be better understood, and specific genes more readily identified, by the discovery of biological characteristics that lie a step closer to DNA than the clinically observable symptoms and signs, the ‘exophenotypes’, by which disorders are defined. Irv continued to elaborate the endophenotype concept over ensuing years and it provoked thousands of papers by others, a sort of Higgs boson for biological psychiatry. Unlike the Higgs particle, the existence of endophenotypes has yet to be proved experimentally for any of the major disorders.

Irv was born in Cleveland, Ohio, to Hungarian-Romanian Jewish emigre parents, Bernard, an insurance agent, and Virginia (nee Weitzner). He was a science enthusiast from an early age and began a physics degree while serving as an officer in the US navy, later switching to psychology. He completed his PhD at the University of Minnesota on the genetics of personality but initially had great difficulty in getting his findings published because of the prevailing orthodoxy in US academia in the late 1950s that behaviour was entirely due to nurture and nothing to do with nature.

After his postdoctoral fellowship in London, Irv returned in 1966 to the biology-friendly department of psychology in Minneapolis and set up one of the first behaviour genetics training programmes in the US. He thereafter held chairs in Washington University in St Louis (1980–85), where I first came under his mentorship as a visiting MRC fellow, and at the University of Virginia (1986–2001), where he set up a clinical psychology doctorate, before returning to Minnesota, where he remained for the rest of his career.

He won many plaudits and prizes worldwide but retained particular affection for and gratitude to the UK, where his recent awards included honorary fellowship of the Royal College of Psychiatrists and King's College London.

Irv is survived by his wife, Carol (nee Applen), whom he married in 1970, and their sons, Adam and David, and grandchildren, Josh, Ava and Fiona.

Irving Isadore Gottesman, clinical psychologist and geneticist, born 29 December 1930; died 29 June 2016

